# Supplementary material for: Toward Spatial Identities in Human Brain Organoids-on-Chip Induced by Morphogen-Soaked Beads
Source: Bioengineering (Basel). 2020 Dec 18;7(4):164. doi: 10.3390/bioengineering7040164 (PMC7766968; doi:10.3390/bioengineering7040164)
Supplement: Supplementary file 1 [file bioengineering-07-00164-s001.pdf]

Supplementary Figures and Tables

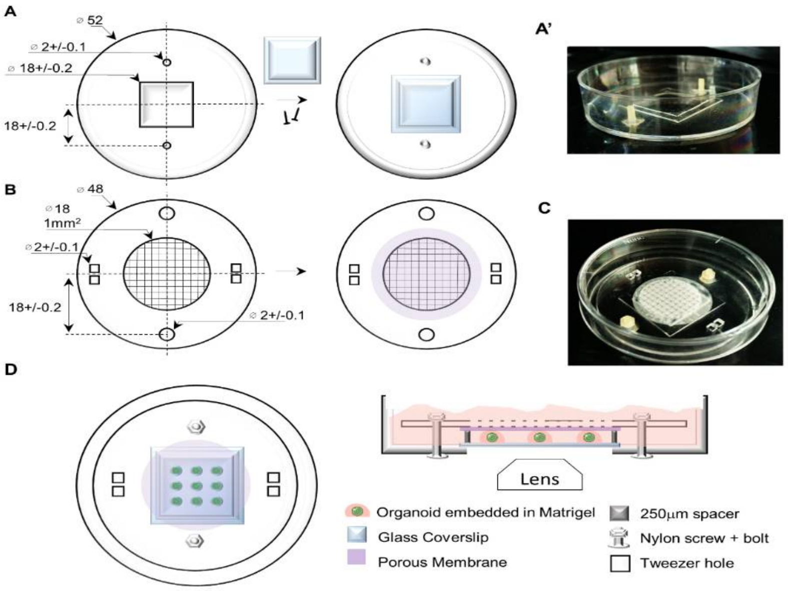

Figure 1. On-chip device assembly. (A) A standard 5cm tissue-culture plate is drilled with 3 holes: 2 of r = 2mm for screws and a r = 18mm² hole for a coverslip. The coverslip is supplied with a 150-250um spacer. Two nylon screws are inserted and glued from the outer part of the plate. **(A')** A picture of the tissue culture plate with the glass coverslip and the nylon screws. **(B)** The plastic insert unit is produced by a laser-cut printer and has a 1x1mm² grid, two r=2mm holes for screws (round) and two pairs of r=2mm holes for tweezers (squared). A porous membrane covers the grid: (a) to allow efficient exchange of nutrients without exposing the tissue and (b) for sealing of the compartment. When assembled, the grid insert seals the compartment and limits the growth of the tissue at the Z axis. The lateral dimensions (X,Y) are not limited. This allows imaging of biological regions which are usually deep within the tissue. This unit is removable and is held by two bolts. **(C)** The assembled device, consisted of a modified tissue-culture plate, a removable grid insert and a lid. **(D)** An illustration showing a top view (left) and a side view (right) of the device loaded with organoids. All measurements in the illustrations are in millimeters.

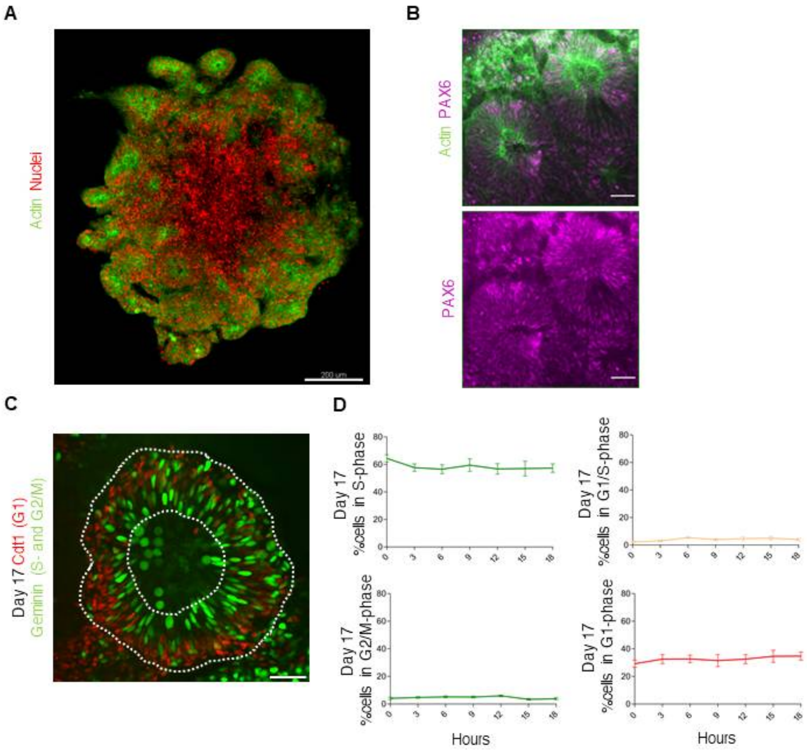

Figure S2. Live-Imaging of Neuroepithelium Tissues Derived from hESCs. (A) Fluorescent image showing an example for brain organoid which was grown on-chip for 18 days. The organoid is observed by live imaging with fluorescent markers of Actin (green) and Nuclei (red). Scale bar 200 m. **(B)** Magnified NE domains of day 18 organoid showing Actin (green) and immunohistochemistry of PAX6 antibody (magenta). Scale bar 50 m. **(C)** Fluorescent image taken from an 18-hrs time-lapse movie of a day 17 organoid showing a NE domain labeled with FUCCI markers (Geminin-GFP, Cdt1-Red). Scale bar 50 m. **(D)** The averaged percentage of cells that were in different cell cycle phases through time: S-phase; G1 to S-phase transition; G2- to M-phase transition; and G1-phase. N=6 Neuroepithelium domains, sampled at 7 timepoints in 3 hrs interval. Error bars represent ±SEM.

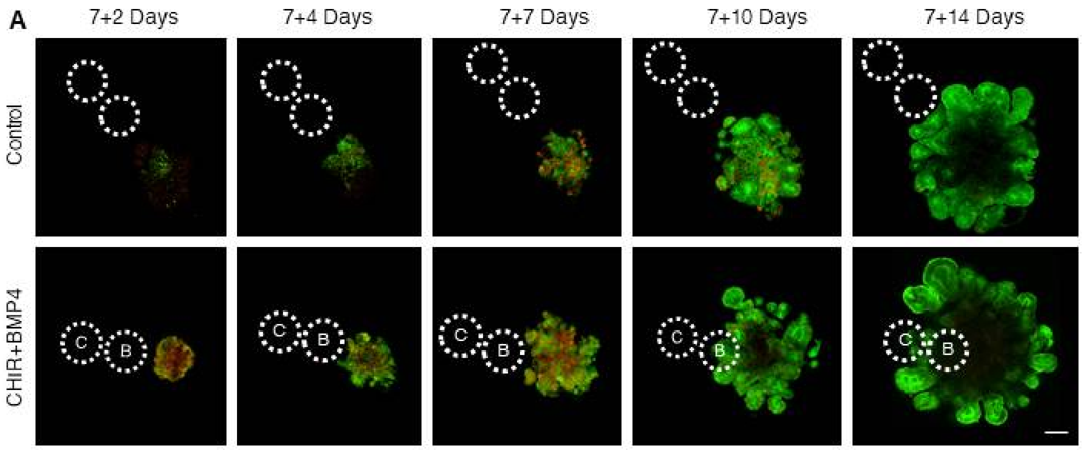

Figure S3. Time-lapse fluorescent images of 1x condition. (A) Extended panel for Figure 3B. Time-lapse fluorescent images showing Actin (green) and Nuclei (red) of a developing control and a 1x condition organoid. Scale bar 200mm.

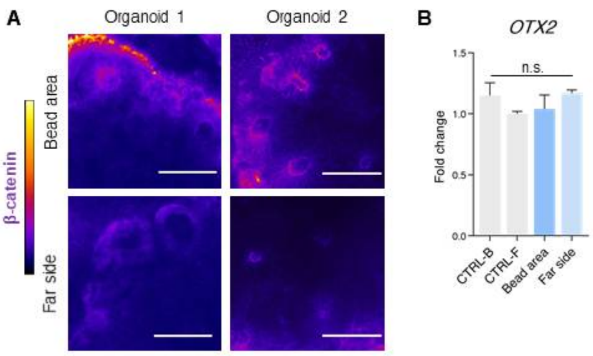

Figure S4. Additional immunohistochemistry and qPCR for 1x condition. (A) Additional examples of immunohistochemistry and heatmap of beta-catenin expression (yellow-high; blue-low) at day 11 of the 1x condition, showing the bead area and the far side. **(B)** qPCR analysis of the changes in *OTX2* gene expression using the 1x condition. Error bars represent ±SEM. N=24 organoids per experimental group. Comparisons were analyzed using *ANOVA* with post-hoc Tukey's multiple comparisons test (DF=20): n.s. non-significant p-value>0.05.

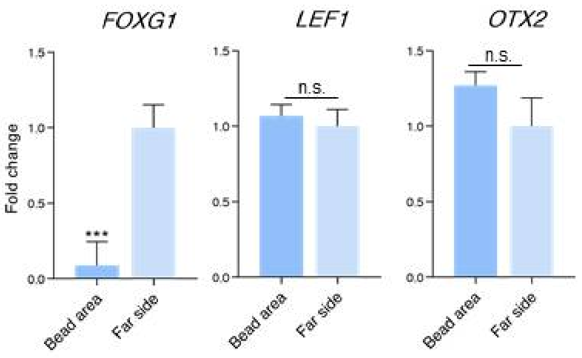

Figure S5. Additional qPCR analysis for 2x condition. qPCR analysis of the gene expression changes in the telencephalon markers: *FOXG1*, *LEF1* and *OTX2* using the 2x condition. Error bars represent ±SEM. N=24 organoids per experimental group. Comparisons were analyzed using *ANOVA* with post-hoc Tukey's multiple comparisons test (DF=15): n.s. non-significant p-value>0.05.

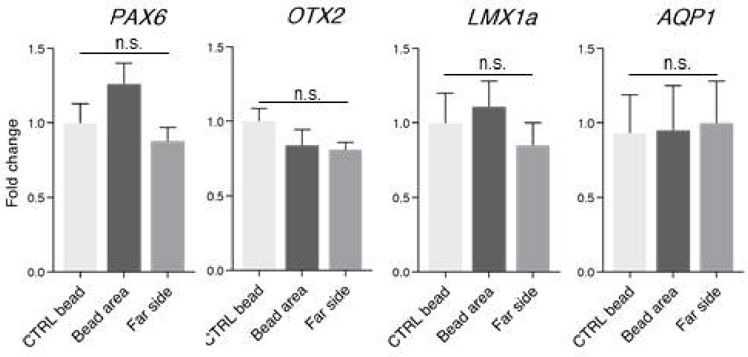

Figure S6. Additional qPCR analysis for 4x condition. qPCR analysis of *PAX6*, *OTX2*, *LMX1a* and *AQP1* genes at the 4x condition. Error bars represent ±SEM. N=24 organoids per experimental group. Comparisons were analyzed using *ANOVA*: n.s. non-significant p-value>0.05.

Table 1. Primer Sequences.

	Forward (5' – 3')	Reverse (5' – 3')	Product Size
AQP1	CTGGGCATCGAGATCATCGG	ATCCCACAGCCAGTGTAGTCA	158bp
EN1	GAGCGCAGGGCACCAAATA	CGAGTCAGTTTTGACCACGG	91bp
EN2	CCGGCGTGGGTCTACTGTA	CCTCTTTGTTCGGGTTCTTCTT	92bp
FGF8	GACCCCTTCGCAAAGCTCAT	CCGTTGCTCTTGGCGATCA	110bp
FOXA2	TTCAGGCCCGGCTAACTCT	AGTCTCGACCCCCACTTGCT	67bp
FOXG1	GCCAGCAGCACTTTGAGTTA	GGTGGAGAAGGAGTGGTTGT	114bp
GAPDH	TCAAGAAGGTGGTGAAGCAG	CGCTGTTGAAGTCAGAGGAG	93bp
GBX2	CTCACCTCTACGCTCATGGC	GCCTTGTCGAAGTTACCGC	125bp
LEF1	TGCCAAATATGAATAACGACCCA	GAGAAAAGTGCTCGTCACTGT	150bp
LMX1a	GCAAAGGGGACTATGAGAAGGA	CGTTTGGGGCGCTTATGGT	160bp
NGN2	AAACCATGTCACGCGCTCA	GCCTTCAGTCTACGGGTCTT	224bp
NKX2.2	AAACCATGTCACGCGCTCA	GGCGTTGTACTGCATGTGCT	111bp
NKX6.1	CACACGAGACCCACTTTTTCC	CCCAACGAATAGGCCAAACG	110bp
OLIG2	GGGCCACAAGTTAGTTGGAA	GAGGAACGGCCACAGTTCTA	110bp
OLIG3	CCTGCTCGCCAGAAACTACA	CCCCATAGATCTCGCCAACC	80bp
OTX2	AGAGGACGACGTTCACTCG	TCGGGCAAGTTGATTTTCAGT	115bp
PAX2	TGTCAGCAAAATCCTGGGCAG	GTCGGGTTCTGTCGTTTGTATT	132bp
PAX6	AGTGCCCGTCCATCTTTGC	CGCTTGGTATGTTATCGTTGGT	81bp
PAX7	ACCCCTGCCTAACCACATC	GCGGCAAAGAATCTTGGAGAC	121bp
WNT1	CGATGGTGGGGTATTGTGAAC	CCGGATTTTGGCGTATCAGAC	133bp
